# Reduced vagal modulations of heart rate during overwintering in Antarctica

**DOI:** 10.1038/s41598-020-78722-3

**Published:** 2020-12-11

**Authors:** Martina A. Maggioni, Giampiero Merati, Paolo Castiglioni, Stefan Mendt, Hanns-Christian Gunga, Alexander C. Stahn

**Affiliations:** 1 Charité – Universitätsmedizin Berlin, Corporate Member of Freie Universität Berlin, Humboldt-Universität zu Berlin, and Berlin Institute of Health, Institute of Physiology, Center for Space Medicine and Extreme Environments Berlin, 10117 Berlin, Germany; 2 Department of Biomedical Sciences for Health, Università degli Studi di Milano, 20133 Milan, Italy; 3 IRCCS Fondazione Don Carlo Gnocchi, 20148 Milan, Italy; 4 Department of Biotechnology and Life Sciences (DBSV), University of Insubria, 21100 Varese, Italy; 5 Department of Psychiatry, Perelman School of Medicine, University of Pennsylvania, 1016 Blockley Hall, 423 Guardian Drive, Philadelphia, PA 19004 USA

**Keywords:** Physiology, Medical research

## Abstract

Long-duration Antarctic expeditions are characterized by isolation, confinement, and extreme environments. Here we describe the time course of cardiac autonomic modulation assessed by heart rate variability (HRV) during 14-month expeditions at the German *Neumayer III* station in Antarctica. Heart rate recordings were acquired in supine position in the morning at rest once before the expedition (baseline) and monthly during the expedition from February to October. The total set comprised twenty-five healthy crewmembers (n = 15 men, 38 ± 6 yrs, n = 10 women, 32 ± 6 yrs, mean ± SD). High frequency (HF) power and the ratio of low to high frequency power (LF/HF) were used as indices of vagal modulation and sympathovagal balance. HF power adjusted for baseline differences decreased significantly during the expedition, indicating a gradual reduction in vagal tone. LF/HF powers ratio progressively shifted toward a sympathetic predominance reaching statistical significance in the final trimester (August to October) relative to the first trimester (February to April). This effect  was particularly pronounced in women. The depression of cardio-vagal tone and the shift toward a sympathetic predominance observed throughout the overwintering suggest a long-term cardiac autonomic modulation in response to isolation and confinement during Antartic overwintering.

## Introduction

Antarctic expeditions have a long history for studying the physiological and psychological effects of isolation, confinement, and extreme environments (ICE)^1^. Except for unprecedented polar traverses in dangerous terrain during the winter, harsh environmental conditions are typically of less concern for modern day expeditions due to significant advances in technology and travel. In contrast, social isolation and confinement associated with Antarctic overwintering can pose considerable psycho-physiological challenges. Prolonged stays in Antarctica have shown to alter circadian^2–4^, immunological^5^, hormonal^6,7^, and neurobehavioral responses including mood disturbances^1,8^ and brain changes^9^. Isolation and confinement are also considered critical stressors during future long-duration spaceflight missions (LDSM) that could put human health and mission success at risk^10^. LDSM and Antarctic expeditions share significant similarities of the psychosocial environment^11^. These include altered day/night cycles, reduced sensory stimulation, sensory monotony, isolation from the outside world, separation from friends and family, absence of privacy, overlap between work and leisure, and exposure to the same small group in both of these settings^1^. Antarctic overwintering has therefore been considered a high-fidelity spaceflight analog for investigating the behavioral risks of isolation and confinement. The neurobehavioral impact of isolation and confinement may vary between and within individuals^12^, depending on previous expedition experience, crew cohesion, professional and social group roles, coping strategies, and sources of social support. Tools for unobtrusively and non-invasively monitoring physiological responses could add to the usefulness of self-reported measures to better understand these phenotypic differences, and support the early detection of any adverse behavioral conditions.

Heart rate variability (HRV) is a measure of cardiac autonomic modulation that has been suggested as a reliable tool to quantify the physiological^13,14^ and neurobehavioral effects of human adaptation to different stressors^15,16^. Greater social integration was shown to be associated with increased high frequency (HF) power of HRV in humans^17^. Data from prairie voles demonstrated significant disruptions of HRV and behavior after isolation from their partner, and these changes were reversed following re-pairing or environmental enrichment^18^. We are aware of only one study that identified the time course of HRV during an Antarctic expedition^19^. This study reported decreases in low frequency (LF) power and in the ratio of LF to HF power at the end of the expedition. However, the small sample (n = 6 men), the limitation of two data collections during the austral summer, and the short study period (about 40 days) warrant a cautious interpretation of the findings. Accordingly, the long-term effects of isolation and confinement on autonomic balance in healthy humans remain to be established. Here, we investigated the time course of short-term HRV during overwintering at the German Neumayer III station in Antarctica. It is the first study that reports findings of the time course during overwintering in Antarctica in both men and women.

## Results

**Figure 1:**
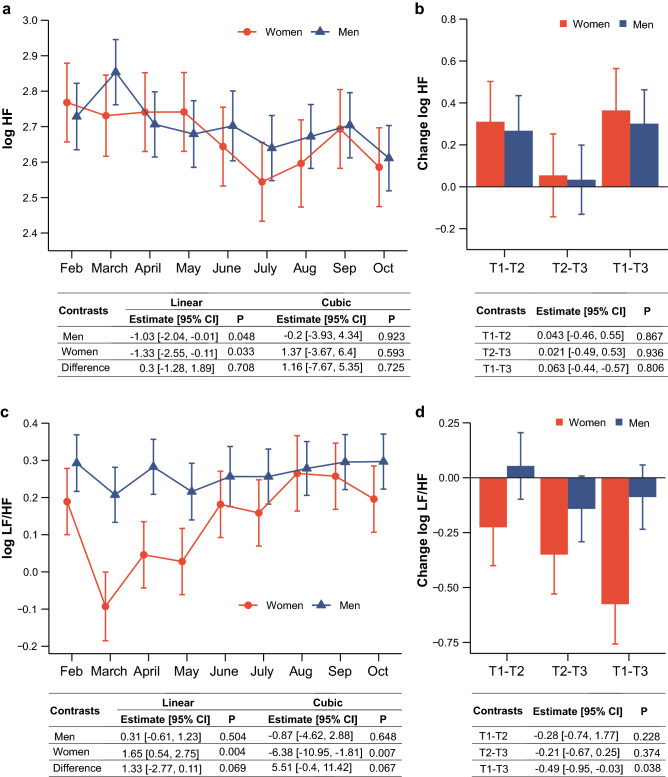
Temporal profiles of log HF (**a**) and log LF/HF (**c**) during Antarctica overwintering in men (blue) and women (red). Data are adjusted means and standard errors. Contrasts show mean effects and 95% CI for linear and cubic trends in men and women and their differences. Detailed polynomial contrast analyses are provided in Table 3. Panel (**b**) and (**d**) show changes between trimesters T1, T2, and T3 for men (blue) and women (red), which were defined as T1: Feb to April; T2: May to July; and T3: Aug to Oct. Contrasts indicate interactions between trimesters and sex, i.e., mean differences between men and women relative to changes in log HF and Log LF/HF from T1 to T2, T2 and T3, and T1 and T3, respectively. Detailed contrasts analyses are reported in Table 5.

Demographic and pre-mission HRV characteristics by sex are shown in Table 1. Women tended to have higher resting HR (*P* = 0.069) and HF power (*P* = 0.070); they also had significantly lower LF/HF ratio than men (*P* = 0.025). At *Neumayer III* station we acquired a total of 225 RR series. Thirteen of these recordings were discarded because of an excessive number of edited beats. Further, an additional eight recordings were identified as outliers and excluded from the final data set (see Statistical analysis). HR in both women and men remained unchanged and did not show trends during the whole period (see Supplementary Table S1). Results from the mixed model analyses are provided in Table 2, showing a significant effect of the expedition on log LF/HF (*P* = 0.02), LF_nu_ (*P* = 0.024), and HF_nu_ (*P* = 0.023). The adjusted means and standard errors for each month and sex are provided in Supplementary Tables S1 and S2. Our pre-defined contrasts revealed a significant effect of the expedition on log HF (*P* < 0.01), which was characterized by a linear decrease in both men and women (linear trend: *P* = 0.033 and *P* = 0.048 for women and men) (Fig. 1a). Visual inspection of the data suggested a slight increase of log HF towards the end of the mission. Nonetheless, neither non-linear trends nor the comparison between trimesters (i.e., T1: Feb to April; T2: May to July; T3: August to October) could confirm such a pattern. As indicated in Fig. 1b, log HF was considerably lower during trimester 2 and 3, and this temporal profile was similar for men and women, suggesting a decrease in vagal activity throughout the mission (see also Table 5). In contrast, log LF showed a different time course between men and women that was qualified by a nonlinear trajectory in women compared to men (cubic trend: *P* = 0.012 for women, and *P* = 0.699 for men; interaction: *P* = 0.089; see also Table 3). As for log LF/HF, the significant shift toward sympathetic predominance during the overwintering was largely driven by women (Fig. 1c, d). Polynomial contrasts confirmed significant linear (LF_nu_, HF_nu_, log LF/HF), and significant cubic (log LF/HF) trends for women, but not men (Table 3 and 4). Likewise, log LF/HF was significantly higher at T3 compared to T1 in women (Table 5). We acknowledge that the differences in error probabilities do not imply statistical differences, but raise the point towards potential sex-specific differences, which is also supported by the nearly significant interactions between sex and time for the linear and cubic trends observed for log LF/HF (*P* = 0.069 and *P* = 0.067).

**Table 1:** General characteristics of the enrolled subjects at baseline.

Variable	Women (N = 10)	Men (N = 15)	*P*
Age (yrs)	31.8 (5.7)	38.4 (8.9)	0.050
Body Mass Index (kg/m^2^)	24.7 (3.6)	26.9 (3.5)	0.143
HR (bpm)	70.3 (6.3)	64.2 (8.5)	0.069
log LF/HF	0.004 (0.379)	0.334 (0.239)	0.025
log HF (ms^2^)	2.824 (0.393)	2.534 (0.266)	0.070

Note: mean (SD); HRV, heart rate variability; *P* values refer to unpaired Student’s *t*-tests comparing men and women.

**Table 2:** Mixed models investigating the effects of expedition duration and sex on heart rate and indices of cardiac autonomic modulation.

Variable	Fixed Effect	*df*_1_	*df*_2_	*F*	*P*
HR	Time	8.00	167.27	0.71	0.682
	Sex	1.00	21.96	0.35	0.561
	Time × Sex	8.00	167.27	0.54	0.827
log HF	Time	8.00	167.33	1.70	0.102
	Sex	1.00	21.81	0.06	0.804
	Time × Sex	8.00	167.33	0.39	0.927
log LF	Time	8.00	167.51	1.85	0.072
	Sex	1.00	21.98	2.25	0.148
	Time × Sex	8.00	167.50	1.49	0.164
log LF/HF	Time	8.00	167.28	2.35	0.020
	Sex	1.00	21.30	2.53	0.127
	Time × Sex	8.00	167.28	0.97	0.464
LF_nu_	Time	8.00	167.23	2.28	0.024
	Sex	1.00	21.24	1.83	0.191
	Time × Sex	8.00	167.23	0.90	0.518
HF_nu_	Time	8.00	167.23	2.29	0.023
	Sex	1.00	21.24	1.81	0.192
	Time × Sex	8.00	167.23	0.91	0.512

Note: Mixed models were run with time and sex as fixed effects, baseline as a covariate, and participants as a random factor (random intercept only); *df*_1_, numerator degrees of freedom; *df*_2_, denominator degrees of freedom; *F*, F-statistic; *P*, *p* value.

**Table 3:** Contrasts for assessing linear, quadratic, and cubic trends of expedition on heart rate and indices of cardiac autonomic modulation in men and women.

Variable	Contrast		Estimate	SE	*df*	*t*	*P*
HR	Linear	Women	9.33	14.33	167.2	0.65	0.516
		Men	15.41	11.95	167.2	1.29	0.199
		Difference	6.08	18.66	167.2	0.33	0.745
	Quadratic	Women	47.33	95.59	167.0	0.50	0.621
		Men	− 8.95	83.32	167.3	− 0.11	0.915
		Difference	− 56.28	126.81	167.1	− 0.44	0.658
	Cubic	Women	− 8.00	59.21	167.3	− 0.14	0.893
		Men	79.24	48.63	167.6	1.63	0.105
		Difference	87.24	76.62	167.4	1.14	0.257
log HF	Linear	Women	− 1.33	0.62	167.2	− 2.15	0.033
		Men	− 1.03	0.51	167.3	− 1.99	0.048
		Difference	0.30	0.80	167.2	0.37	0.708
	Quadratic	Women	2.44	4.12	167.0	0.59	0.554
		Men	0.92	3.59	167.4	0.26	0.798
		Difference	− 1.52	5.46	167.2	− 0.28	0.781
	Cubic	Women	1.36	2.55	167.3	0.54	0.593
		Men	0.20	2.09	167.8	0.10	0.923
		Difference	− 1.16	3.30	167.5	− 0.35	0.725
log LF	Linear	Women	0.47	0.57	167.4	0.82	0.415
		Men	− 0.72	0.48	167.4	− 1.51	0.134
		Difference	− 1.19	0.75	167.4	− 1.59	0.113
	Quadratic	Women	5.72	3.82	167.1	1.50	0.136
		Men	3.19	3.33	167.5	0.96	0.339
		Difference	− 2.53	5.07	167.3	− 0.50	0.618
	Cubic	Women	− 5.99	2.37	167.5	− 2.53	0.012
		Men	− 0.75	1.94	168.0	− 0.39	0.699
		Difference	5.24	3.06	167.7	1.71	0.089
log LF/HF	Linear	Women	1.65	0.56	167.1	2.94	< 0.01
		Men	0.31	0.47	167.2	0.67	0.504
		Difference	− 1.33	0.73	167.1	− 1.83	0.069
	Quadratic	Women	2.64	3.74	166.7	0.71	0.481
		Men	2.38	3.26	167.3	0.73	0.466
		Difference	− 0.26	4.96	167.0	− 0.05	0.958
	Cubic	Women	− 6.38	2.31	167.3	− 2.76	< 0.01
		Men	− 0.87	1.90	168.1	− 0.46	0.648
		Difference	5.51	2.99	167.6	1.84	0.067

Note: SE, standard error; *df*, degrees of freedom, *t*, t-statistic for parameter estimate; *P*, p value.

**Table 4:** Contrasts for assessing linear, quadratic, and cubic trends of expedition on normalized units indices of cardiac autonomic modulation in men and women.

Variable	Contrast		Estimate	SE	*df*	*t*	*P*
LF_nu_	Linear	Women	74.68	26.77	167.0	2.79	< 0.01
		Men	8.65	22.33	167.1	0.39	0.699
		Difference	− 66.03	34.86	167.1	− 1.89	0.060
	Quadratic	Women	136.29	178.66	166.7	0.76	0.447
		Men	103.71	155.65	167.3	0.67	0.506
		Difference	− 32.58	236.95	167.0	− 0.14	0.891
	Cubic	Women	− 276.03	110.62	167.2	− 2.50	0.014
		Men	− 52.68	90.78	168.0	− 0.58	0.563
		Difference	223.36	143.10	167.5	1.56	0.120
HF_nu_	Linear	Women	− 74.99	26.74	167.0	− 2.80	< 0.01
		Men	− 8.55	22.31	167.1	− 0.38	0.702
		Difference	66.44	34.82	167.1	1.91	0.058
	Quadratic	Women	− 137.89	178.49	166.7	− 0.77	0.441
		Men	− 104.67	155.50	167.3	− 0.67	0.502
		Difference	33.22	236.72	167.0	0.14	0.889
	Cubic	Women	277.24	110.51	167.2	2.51	0.013
		Men	52.71	90.69	168.0	0.58	0.562
		Difference	− 224.53	142.96	167.5	− 1.57	0.118

Note: SE, standard error; df, degrees of freedom, *t*, t-statistic for parameter estimate; *P*, *p* value.

**Table 5:** Contrasts assessing changes between trimesters.Note: SE, standard error; df, degrees of freedom, *t*, t-statistic for parameter estimate; *P*, *p* value.

Variable	Contrast		Estimate	SE	*df*	*t*	*P*
log HF	T1–T2	Women	0.31	0.19	167.0	1.61	0.108
		Men	0.26	0.16	167.3	1.60	0.112
		Difference	0.04	0.255	167.178	0.16	0.867
	T2–T3	Women	0.05	0.19	167.2	0.27	0.784
		Men	0.03	0.16	167.1	0.20	0.839
		Difference	0.02	0.25	167.2	0.08	0.936
	T1–T3	Women	0.36	0.20	167.4	1.82	0.070
		Men	0.30	0.16	167.2	1.86	0.064
		Difference	0.06	0.25	167.3	0.24	0.806
log LF/HF	T1–T2	Women	− 0.22	0.17	166.7	− 1.29	0.197
		Men	0.05	0.15	167.3	0.35	0.723
		Difference	− 0.28	0.23	167.0	− 1.21	0.228
	T2–T3	Women	− 0.35	0.17	167.1	− 1.95	0.053
		Men	− 0.14	0.15	167.0	− 0.94	0.345
		Difference	− 0.20	0.23	167.1	− 0.89	0.374
	T1–T3	Women	− 0.57	0.18	167.4	− 3.17	< 0.01
		Men	− 0.08	0.14	167.0	− 0.60	0.549
		Difference	− 0.48	0.23	167.2	− 2.09	0.038

## Discussion

We investigated the time course of cardiac autonomic modulation using HRV during overwintering at the German *Neumayer III* station in Antarctica. We found a significant gradual decrease in parasympathetic tone that is somewhat plateauing after about two thirds of the mission, and a concomitant shift towards sympathetic predominance. These results seem to contradict previous data demonstrating a reduced day-time sympathovagal balance at the end of a 40-day stay on the Italian *Zucchelli* station at Terra Nova Bay^19^. The two data sets vary significantly with respect to their study design and sample size: (1) the group size residing at the station comprised at total of n = 75 persons at *Zucchelli* vs. n = 9 crewmembers at *Neumayer III* station; (2) the present study investigated the time course of HRV over 9 months compared to two measurements at *Zucchelli* station collected within less than two months during the austral summer; the expedition duration comprised 14 months at *Neumayer III* station vs. about 1.5 months at *Zucchelli* station. (3) Finally, data at *Zucchelli* station were collected in n = 6 men compared to a total of n = 25 (n = 10 women) at *Neumayer III* station. Our data suggest distinct differences in sympathovagal balance (LF/HF ratio), which has also been shown in recent meta-analyses^20^ .

Nonetheless, it is too simplistic to attribute the discrepancy of the findings to sex-specific differences because the gradual linear decrease in cardiac vagal drive throughout the expedition was consistent for men and women. For instance, data from isolation studies targeted at small crews and longer study periods, i.e., 105 days and 520 days of isolation also showed an increase of parasympathetic activity during wakefulness (HF_nu_ increased), and diminished parasympathetic activity during sleep periods (HF_nu_ decreased)^21,22^. The authors speculated that the diminished circadian rhythm of the autonomic cardiac modulation could be related to a variety of factors including monotony and boredom and a lack of daylight exposure during isolation and confinement^22^.

Light is the primary external cue for entraining the circadian system, and plays a key role for regulating the sleep–wake rhythms of the autonomic nervous system^23^. Recent research shows that solar activity also affects HRV. It was shown that solar radio flux was positively associated with HF power^24^. The role of solar activity is somewhat supported by the time course of log HF in women in the present study. The time course of log HF in women was characterized by a significant cubic trend (*P* = 0.012). Following a relatively stable profile during the first four months, log HF power linearly decreased between May and July, and then increased between July and September concomitant with considerable changes in sunshine duration at *Neumayer III* station^25^. The slight reduction in log HF power during the final data recording in October remains speculative. A growing body of research highlights the role of cardiac vagal control in regulating emotional and stress responses^26^. It is possible that the crew is starting their preparation for the austral summer, which is characterized by a considerable increase in operational and logistical activities, and external researchers and staff visiting the station. Likewise, the marked decrease in log LF/HF in March could reflect the psycho-physiological response to the end of the demanding summer period characterized by extensive working hours, reduced privacy due to the increased number of staff and researchers, and the pressure to successfully take over the station and expectations to function as a team in an unknown operational environment.

## Strengths and limitations

According to the authors’ best knowledge, this is the first study investigating the time course during long-duration overwintering in Antarctica. We collected monthly recordings of HRV in the morning at rest over a series of three overwintering campaigns, resulting in a total sample size of 25 participants comprising men and women.

However, we also acknowledge several limitations. First, our analysis focused on short-term recordings of HRV acquired in the morning, because they were reported to be representative of the autonomic modulations of HR^27^. Thus, we cannot verify any differences in autonomic modulation during different daily activities and sleep and circadian changes in response to long-duration Antarctic overwintering.

Second, we did not control our analyses for addtional factors that have also been shown to affect HRV such as changes in hydration status or body weight, sleep quality, or environmental factors such as the sunshine duration, geomagnetic activity, and cosmic radiation^24^.

Third, we did not directly record the respiratory frequency. Changes in respiratory rate over time could potentially contribute to the observed changes in the HF power. Given that *Neumayer III* station is located at sea level we may safely exclude respiratory effects observed in response to high altitude exposure. Moreover, to mitigate the possible effects of different respiratory patterns we standardized the data collections (lying position, same time of the day for all sessions), and removed HF-power outliers from the final data set (see Statistical analysis). We are confident that these procedures minimized confounding effects associated with irregular breathing patterns. In line with that, the frequency of the highest spectral peak in the HF band (see Supplementary Table S3), providing  indirect evidence of the central respiratory frequency, was in the physiological range for healthy adults, and remarkably stable in men and women during the entire expedition.

Furthermore, we did not quantify behavioral responses as self-reported measures of social isolation, emotional deprivation, and stress levels, which would highly valuable to better understand phenotypic differences in HRV responses relative to coping strategies^15^. Likewise, we cannot infer the physiologic mechanisms responsible for our findings because we did not correlate our data with neuroimmunologic and hormonal responses. Finally, our data revealed characteristic differences in the time course of HRV indices between men and women that require further studies to elucidiate potential sex-related differences in HRV responses to prolonged isolation and confinement.

Taken together, this study found a phenomenon of cardiac autonomic modulation that has not been observed in other ICC and ICE analogs: a persistent vagal modulation depression throughout the expedition that is particularly marked during the second trimester of the expedition. This finding is particularly striking as it contrasts previous research investigating shorter stays in Antarctica^19^ and long-duration isolation studies in ICCs^21,22^. Visual inspection of the data suggested that the change in autonomic cardiac modulation was more pronounced in women. Larger studies collecting 24-h ECG recordings throughout the entire expedition are needed to detect sex-specific differences and determine the circadian and circannual rhythms of the autonomous nervous system during long-duration Antarctic overwintering. Combining HRV recordings with a set of behavioral and environmental measures will allow for an integrative understanding of the effects of social isolation, group size and dynamics, and sunshine duration and geomagnetic activity on HRV. Such approaches will help to verify the use of HRV as a non-invasive tool to assess individual differences in self-regulatory coping strategies in response to isolation and confinement associated with exploratory class spaceflight missions or conditions of large-scale social restrictions such as during the COVID-19 pandemic.

## Methods

### Participants

Participants were recruited from three consecutive winter-over expedition crews (each n = 9) staying at the German *Neumayer III* station for 14 months. A total of twenty-five healthy participants (men: n = 15, 38 ± 6 yrs, 87 ± 10 kg, 180 ± 6 cm; women: n = 10, 32 ± 6 yrs, 68 ± 6 kg, 166 ± 6 cm; mean ± SD) were enrolled in the study (Mission 1, n = 9, Mission 2, n = 7, and Mission 3, n = 9). Each expedition was preceded by a 4.5-month extensive operational training program including general education and training about running and living on *Neumayer III* station, and specific professional training related to different operational responsibilities. Following the training program the crews departed to Antarctica in December, and the station was handed over from the previous crew, which then returned to Europe typically around the end of January/beginning of February. A detailed description of *Neumayer III* station and operational characteristics is provided elsewhere^9^. Subjects provided written informed consent to participate in the study, which was approved by the local Ethic Committee of the Charité – Universitätsmedizin Berlin, Berlin, Germany. All experimental sessions were performed in full accordance with the principles of the Declaration of Helsinki^28^.

### Experimental procedures

Data were collected in the morning between 9.00 and 12.00 a.m. in supine position at rest using a mobile heart rate (HR) monitor (Polar S810, Polar Electro Oy, Kempele, Finland) that provides beat-by-beat time series of RR intervals with resolution of 1 ms^29^. A baseline recording was performed about two months before the departure to Antarctica at Charité – Universitätsmedizin Berlin, including a 12-lead resting electrocardiogram (ECG) to check sinus rhythm and exclude arrhythmias. The baseline session was followed by a training session on how to operate the HR monitor and perform the recording according to a strict procedure. All data were collected in supine position for 10 min, and participants were instructed to refrain from caffeine consumption for at least three hours before the measurement and limiting any strenuous exercise 24 h prior to the data recording. In-mission data were collected monthly from February to October, resulting in a total of 250 h recordings.

### HRV data pre-processing and analysis

Normal-to-normal (NN) interval series were obtained by visual inspection of each RR series, removing possible premature beats and artefacts^14^. The entire series was discarded if the number of artefacts or ectopic beats exceeded 5% of all RR intervals. HRV was quantified by indices in frequency domain^14^. The power spectrum of NN intervals was calculated from the Welch periodogram, deriving spectral powers in the low-frequency (LF, from 0.04 to 0.15 Hz) and high-frequency (HF, from 0.15 to 0.40 Hz) bands^14^. The HF power comprises respiratory oscillations mediated by the cardiac vagal drive, whereas the LF/HF powers ratio is an index of cardiac sympathovagal balance^14,30,31^. Furthermore LF/HF ratio is an HRV index sensitive to ongoing stress, as previously reported^32,33^. HF, LF and LF/HF indices were log transformed for further analyses. LF and HF were also expressed as normalized units (nu) as follows: HF_nu_ = HF/(total power—VLF) × 100 and LF_nu_ = LF/(total power—VLF) × 100, were the VLF is the spectral power in the very low-frequency band (0–0.04 Hz). The LF_nu_ is considered an index of sympathovagal balance, the HF_nu_ an index of vago/sympathetic balance. The indices were calculated with the software Kubios HRV ver. 2.2 (Kuopio, Finland)^34^.

### Statistical analysis

Anthropometric, HR, log HF and log LF/HF differences between men and women at baseline were assessed using unpaired Student’s *t* tests, before the expedition. None of the baseline recordings was discarded because of premature beats or artifacts, while 13 out of 225 recordings performed in Antarctica were discarded because of an excessive number of edited beats.

Eight additionally monthly recordings were excluded because identified as outliers. Unlike pre-mission recordings, obtained in a controlled laboratory under supervision, recordings in Antarctica were performed by the volunteers themselves. Thus we checked these recordings for the possible presence of outliers before applying any statistical test. Considering that the vagal tone is particularly sensitive to external perturbations, changes in breathing patterns, or lack of steady-state conditions, outliers were identified with Tukey’s method^35^ applied on the index of vagal HR modulations, log HF. For each volunteer we considered the distribution of 9 monthly estimates in Antarctica of log HF, calculated the first and third quartiles (Q1 and Q3) of the distribution, and identified values of log HF as outliers, if they were smaller than Q1─1.5 × (Q3–Q1) or larger than Q3 + 1.5 × (Q3–Q1). When a log HF estimate was identified as an outlier, we discarded the corresponding HR recording.

To analyze the time courses of HRV we first formulated mixed models with subject as a random factor, time and sex as fixed factors, and baseline data (data collected before the expedition) as a covariate. Variance components were estimated using restricted maximum likelihood (REML) approach using the R package lme4^36^. Normality and homogeneity were checked by visual inspection of plots of residuals against fitted values (Q–Q plots). Next, the adjusted means (estimated marginal means) were used to assess the linear, quadratic, cubic trends for each sex, and determined their interaction using pre-planned polynomial contrasts. We also defined custom contrasts averaging the data across trimesters (T1, T2, and T3) and compared the changes in HRV between trimesters (T1–T2, T2–T3, and T1–T3) to further elucidate sex differences. No corrections of the level of significance were applied^37^. We acknowledge that this increased the chance of Type I errors. However, given the exploratory nature of the study, we were also cautious about false negatives^38^. As a compromise we limited the tests to a small set of pre-planned contrasts and kept the level of significance at α = 0.05 (two-sided) for all testing. Note that whereas we also provide *P*-values for the fixed effects of the mixed models using Satterthwaite’s approximation for denominator degrees of freedom^39^, our primary hypotheses focus on the estimates related to our a priori defined linear, quadratic, cubic^40^ . All statistical analyses were carried out using the software package R^41^.

## Supplementary information

Supplementary Information.

## Data Availability

The data that support the findings of this study are openly available in *figshare* at. https://doi.org/10.6084/m9.figshare.12901682
