# Supplementary material for: Reduced vagal modulations of heart rate during overwintering in Antarctica
Source: Sci Rep. 2020 Dec 11;10:21810. doi: 10.1038/s41598-020-78722-3 (PMC7733485; doi:10.1038/s41598-020-78722-3)
Supplement: Supplementary file 1 — Supplementary Information. [file 41598_2020_78722_MOESM1_ESM.docx]

**Supplementary Information**

**REDUCED VAGAL MODULATIONS OF HEART RATE**

**DURING OVERWINTERING IN ANTARCTICA**

Maggioni Martina A.^1,2*^, Merati Giampiero^2,3^, Castiglioni Paolo^3^, Mendt Stefan^1^,

Gunga Hanns-Christian^1^, Stahn Alexander C.^1,4*^

^1^ Charité – Universitätsmedizin Berlin, Corporate Member of Freie Universität Berlin, Humboldt-Universität zu Berlin, and Berlin Institute of Health, Institute of Physiology, Center for Space Medicine and Extreme Environments Berlin; Berlin 10117, Germany

^2^ Department of Biomedical Sciences for Health, Università degli Studi di Milano; Milan, 20133, Italy.

^3^ IRCCS Fondazione Don C. Gnocchi; Milan, 20148, Italy.

^4^ Department of Psychiatry, Perelman School of Medicine, University of Pennsylvania, 1016 Blockley Hall, 423 Guardian Drive, Philadelphia, PA 19004, USA

*Corresponding Authors:

Martina A. Maggioni ([martina.maggioni@charite.de](mailto:martina.maggioni@charite.de)) and

Alexander C. Stahn ([astahn@pennmedicine.upenn.edu](mailto:alexander.stahn@charite.de))

**Table S1.** Heart rate and indices of cardiac autonomic modulation during Antarctic expedition in men and women.

| **Variable** | **Time** |  | **Women** | |  |  | **Men** | |
| --- | --- | --- | --- | --- | --- | --- | --- | --- |
|  |  | **n** | **Mean** | **SE** |  | **n** | **Mean** | **SE** |
| **HR** | Feb | 10 | 67.48 | 2.82 |  | 13 | 64.46 | 2.38 |
|  | Mar | 9 | 69.00 | 2.90 |  | 14 | 64.61 | 2.33 |
|  | Apr | 10 | 67.39 | 2.82 |  | 14 | 68.44 | 2.33 |
|  | May | 10 | 65.75 | 2.82 |  | 13 | 66.35 | 2.37 |
|  | Jun | 10 | 67.91 | 2.82 |  | 11 | 65.58 | 2.48 |
|  | Jul | 10 | 69.77 | 2.82 |  | 14 | 66.79 | 2.33 |
|  | Aug | 7 | 67.74 | 3.09 |  | 14 | 65.50 | 2.29 |
|  | Sep | 10 | 68.05 | 2.82 |  | 14 | 66.21 | 2.33 |
|  | Oct | 10 | 69.34 | 2.82 |  | 15 | 68.47 | 2.33 |
| **log HF** | Feb | 10 | 2.77 | 0.11 |  | 13 | 2.73 | 0.09 |
|  | Mar | 9 | 2.73 | 0.11 |  | 14 | 2.85 | 0.09 |
|  | Apr | 10 | 2.74 | 0.11 |  | 14 | 2.71 | 0.09 |
|  | May | 10 | 2.74 | 0.11 |  | 13 | 2.68 | 0.09 |
|  | Jun | 10 | 2.64 | 0.11 |  | 11 | 2.70 | 0.10 |
|  | Jul | 10 | 2.54 | 0.11 |  | 14 | 2.64 | 0.09 |
|  | Aug | 7 | 2.60 | 0.12 |  | 14 | 2.67 | 0.09 |
|  | Sep | 10 | 2.69 | 0.11 |  | 14 | 2.70 | 0.09 |
|  | Oct | 10 | 2.59 | 0.11 |  | 15 | 2.61 | 0.09 |
| **log LF** | Feb | 10 | 2.98 | 0.10 |  | 13 | 3.01 | 0.08 |
|  | Mar | 9 | 2.66 | 0.10 |  | 14 | 3.04 | 0.08 |
|  | Apr | 10 | 2.73 | 0.10 |  | 14 | 2.98 | 0.08 |
|  | May | 10 | 2.79 | 0.10 |  | 13 | 2.88 | 0.08 |
|  | Jun | 10 | 2.84 | 0.10 |  | 11 | 2.95 | 0.09 |
|  | Jul | 10 | 2.72 | 0.10 |  | 14 | 2.88 | 0.08 |
|  | Aug | 7 | 2.88 | 0.11 |  | 14 | 2.94 | 0.08 |
|  | Sep | 10 | 2.97 | 0.10 |  | 14 | 2.99 | 0.08 |
|  | Oct | 10 | 2.80 | 0.10 |  | 15 | 2.89 | 0.08 |
| **log LF/HF** | Feb | 10 | 0.19 | 0.09 |  | 13 | 0.29 | 0.08 |
|  | Mar | 9 | -0.09 | 0.09 |  | 14 | 0.21 | 0.07 |
|  | Apr | 10 | 0.05 | 0.09 |  | 14 | 0.28 | 0.07 |
|  | May | 10 | 0.03 | 0.09 |  | 13 | 0.22 | 0.08 |
|  | Jun | 10 | 0.18 | 0.09 |  | 11 | 0.26 | 0.08 |
|  | Jul | 10 | 0.16 | 0.09 |  | 14 | 0.26 | 0.07 |
|  | Aug | 7 | 0.27 | 0.10 |  | 14 | 0.28 | 0.07 |
|  | Sep | 10 | 0.26 | 0.09 |  | 14 | 0.30 | 0.07 |
|  | Oct | 10 | 0.20 | 0.09 |  | 15 | 0.30 | 0.07 |

Note: Mean, adjusted means; SE, standard errors; n, number of data sets per measurement.

**Table S2.** Normalized units indices of cardiac autonomic modulation during Antarctic expedition in men and women.

| **Variable** | **Time** |  | **Women** | |  |  | **Men** | |
| --- | --- | --- | --- | --- | --- | --- | --- | --- |
|  |  | **n** | **Mean** | **SE** |  | **n** | **Mean** | **SE** |
| **LF_nu_** | Feb | 10 | 60.11 | 4.33 |  | 13 | 64.72 | 3.68 |
|  | Mar | 9 | 46.38 | 4.48 |  | 14 | 60.32 | 3.59 |
|  | Apr | 10 | 54.03 | 4.33 |  | 14 | 63.88 | 3.59 |
|  | May | 10 | 52.17 | 4.33 |  | 13 | 60.39 | 3.69 |
|  | Jun | 10 | 59.66 | 4.33 |  | 11 | 63.26 | 3.91 |
|  | Jul | 10 | 59.16 | 4.33 |  | 14 | 62.03 | 3.59 |
|  | Aug | 7 | 61.16 | 4.90 |  | 14 | 63.20 | 3.51 |
|  | Sep | 10 | 63.95 | 4.33 |  | 14 | 64.66 | 3.59 |
|  | Oct | 10 | 60.29 | 4.33 |  | 15 | 63.56 | 3.59 |
| **HF_nu_** | Feb | 10 | 39.81 | 4.32 |  | 13 | 35.22 | 3.68 |
|  | Mar | 9 | 53.55 | 4.48 |  | 14 | 39.62 | 3.58 |
|  | Apr | 10 | 45.93 | 4.32 |  | 14 | 36.08 | 3.58 |
|  | May | 10 | 47.79 | 4.32 |  | 13 | 39.58 | 3.68 |
|  | Jun | 10 | 40.28 | 4.32 |  | 11 | 36.70 | 3.90 |
|  | Jul | 10 | 40.80 | 4.32 |  | 14 | 37.92 | 3.58 |
|  | Aug | 7 | 38.67 | 4.89 |  | 14 | 36.77 | 3.50 |
|  | Sep | 10 | 36.00 | 4.32 |  | 14 | 35.30 | 3.58 |
|  | Oct | 10 | 39.60 | 4.32 |  | 15 | 36.39 | 3.58 |

Note: Mean, adjusted means; SE, standard errors; n, number of data sets per measurement.

**Table S3.** Peak frequency of the HF power (Mean and Standard Deviation, SD)

| **Variable** | **Time** |  | **Women** | |  |  | **Men** | |
| --- | --- | --- | --- | --- | --- | --- | --- | --- |
|  |  | **n** | **Mean** | **SD** |  | **n** | **Mean** | **SD** |
| **HF_peak_**  **(Hz)** | Feb | 10 | 0.24 | 0.08 |  | 13 | 0.23 | 0.07 |
|  | Mar | 9 | 0.25 | 0.07 |  | 14 | 0.25 | 0.08 |
|  | Apr | 10 | 0.22 | 0.07 |  | 14 | 0.20 | 0.06 |
|  | May | 10 | 0.24 | 0.06 |  | 13 | 0.22 | 0.07 |
|  | Jun | 10 | 0.21 | 0.06 |  | 11 | 0.23 | 0.06 |
|  | Jul | 10 | 0.25 | 0.07 |  | 14 | 0.22 | 0.06 |
|  | Aug | 7 | 0.21 | 0.08 |  | 14 | 0.22 | 0.07 |
|  | Sep | 10 | 0.23 | 0.07 |  | 14 | 0.22 | 0.07 |
|  | Oct | 10 | 0.21 | 0.05 |  | 15 | 0.23 | 0.07 |
